# Activation of the SOS response increases the frequency of small colony variants

**DOI:** 10.1186/s13104-015-1735-2

**Published:** 2015-12-08

**Authors:** Martin Vestergaard, Wilhelm Paulander, Hanne Ingmer

**Affiliations:** Department of Veterinary Disease Biology, Faculty of Health and Medical Sciences, University of Copenhagen, Stigbøjlen 4, 1870 Frederiksberg C, Denmark

**Keywords:** *Staphylococcus aureus*, Small colony variants, Mutation, SOS response

## Abstract

**Background:**

In *Staphylococcus aureus* sub-populations of slow-growing cells forming small colony variants (SCVs) are associated with persistent and recurrent infections that are difficult to eradicate with antibiotic therapies. In SCVs that are resistant towards aminoglycosides, mutations have been identified in genes encoding components of the respiratory chain. Given the high frequencies of SCVs isolated clinically it is vital to understand the conditions that promote or select for SCVs.

**Results:**

In this study we have examined how exposure to sub-inhibitory concentrations of antibiotics with different mechanism of action influence the formation of SCVs that are resistant to otherwise lethal concentrations of the aminoglycoside, gentamicin. We found that exposure of *S. aureus* to fluoroquinolones and mitomycin C increased the frequency of gentamicin resistant SCVs, while other antibiotic classes failed to do so. The higher proportion of SCVs in cultures exposed to fluoroquinolones and mitomycin C compared to un-exposed cultures correlate with an increased mutation rate monitored by rifampicin resistance and followed induction of the SOS DNA damage response.

**Conclusion:**

Our observations suggest that environmental stimuli, including antimicrobials that reduce replication fidelity, increase the formation of SCVs through activation of the SOS response and thereby potentially promote persistent infections that are difficult to treat.

## Background

The formation of a small colony phenotype compared to wild type colony morphology has been observed in clinical specimens across many bacterial genera comprising Gram-positives and -negatives [1]. Clinically, the emergence of slow-growing *Staphylococcus aureus* mutants, known as small colony variants (SCVs), is associated with sub-acute infections displaying enhanced intracellular residency in host cells promoting persistent and recurrent infections and an increased risk of antibiotic treatment failure [1]. The SCV phenotype can be caused by disruptions of metabolic pathways, conferred either by mutations in the gene *thyA* of the pathway that converts uracil to thymidine [2] or by mutations in the menadione and hemin production pathways resulting in a dysfunctional electron transport chain [3, 4]. Depending on the pathways inactivated, antibiotic resistance to different classes of antimicrobial agents may occur [5]. Mutations in *thyA* confer trimethoprim-sulfamethoxazole resistance [2, 6], while mutations in genes of the biosynthesis pathways encoding menadione or hemin confer resistance to aminoglycosides [3, 4] and certain antimicrobial peptides [7, 8]. The resistance mechanism to aminoglycosides in SCVs defective in the electron transport chain has been linked to a decrease in membrane potential compared to wild type cells, causing a reduction in aminoglycosides uptake [1, 9]. SCVs with a defective electron transport chain exhibit distinct phenotypic characteristics affecting pathogenesis, i.e. reduced pigmentation, decreased hemolysis activity and most often display auxotrophy for menadione and hemin [1]. In a wild type *S. aureus* population a sub-population exists as SCVs [10], however environmental stimuli may increase the frequency of SCV formation. Increased frequency of *S. aureus* gentamicin resistant SCVs has been detected during co-cultures of *S. aureus* and *Pseudomonas aeruginosa* [11], in the intracellular milieu of endothelial cells [12] or in strains with reduced replication fidelity [13]. Short-term enhanced mutation rates of bacterial cells can occur through activation of the SOS response [14] and therefore activation of the SOS response could potentially increase the frequency of SCVs. The SOS regulon is generally repressed by LexA, but upon DNA damage it undergoes autocleavage in response to RecA binding to single-stranded DNA [15]. Inactivation of the LexA repressor leads to the expression of the SOS regulon. This regulon includes genes involved in DNA repair, recombination, as well as error-prone polymerases [16]. The error-prone polymerase V, encoded by the gene *umuC*, is highly up-regulated upon SOS response activation in *S. aureus* [17, 18]. In addition to traditional DNA damaging agents like UV and mitomycin C, other agents also induce the SOS response such as antibiotics of the flouroquinolone class, i.e. ciprofloxacin and moxifloxacin [17, 18]. Therefore we have investigated if antimicrobial compounds that interfere with DNA-, RNA-, protein- and cell wall synthesis affect the proportion of SCVs in the population.

## Results and discussion

To address the effect of the SOS response and exposure to antimicrobials on the formation of gentamicin resistant SCVs in *S. aureus* we employed two strains, the wild type strain 8325-4 as well as an isogenic mutant expressing a non-cleavable LexA variant (*lexA S130A)* that is unable to induce the SOS response [18]. When measuring the minimum inhibitory concentration (MIC) of the strains, increased susceptibility was evident for the *lexA(S130A)* mutant towards chloramphenicol, as well as the DNA targeting antimicrobials; ciprofloxacin, moxifloxacin and mitomycin C, while equal MIC between the two strains was observed for gentamicin, rifampicin and vancomycin (Table 1).

**Table 1:** MICs (μg/ml) of the antimicrobials used in this study for 8325-4 and its *lexA* derivative

Antibiotic	8325-4	*lexA(S130A)*
Gentamicin	1	1
Ciprofloxacin	1/2	1/4
Moxifloxacin	1/16	1/32
Vancomycin	1	1
Chloramphenicol	4	2
Rifampicin	1/512	1/512
Mitomycin C	1/8	1/32

**Fig. 1:**
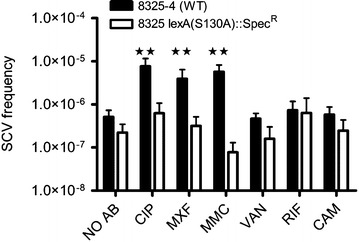
Effect of sub-inhibitory concentrations of antimicrobials on SCV frequency. The frequency of gentamicin resistant SCVs in WT 8325-4 (*black*) population and in the isogenic mutant *lexA(S130A)* (*white*) grown in absence of antibiotics (NO AB) or in the presence of sub-inhibitory concentrations of CIP (Ciprofloxacin), MXF (Moxifloxacin), MMC (Mitomycin C), VAN (Vancomycin), RIF (Rifampicin) and CAM (Chloramphenicol). Concentrations of antimicrobials are 0.5 MIC of the respective strains. The *bars* represent the mean from 8 independent selections of each condition and error bars represent 95 % confidence intervals

Subsequently we measured the effect of sub-inhibitory concentrations (0.5 MIC) of antibiotics on the formation of gentamicin resistant SCVs using the wild type *S. aureus* strain 8235-4 and the isogenic *lexA(S130A)* mutant. The frequency of gentamicin resistant SCVs in an un-exposed culture of 8325-4 was on average 5.1 × 10^−7^, whereas 24 h exposure to mitomycin C, ciprofloxacin and moxifloxacin increased the frequencies of SCVs 11-, 15- and 8-fold, respectively, compared to the SCV frequency of the un-exposed 8325-4 (Fig. 1). The remaining three antimicrobials assayed, chloramphenicol, vancomycin and rifampicin, did not affect SCV formation. For the mutant strain expressing the non-cleavable *lexA(S130A)* exposure to none of the antimicrobials influenced SCV formation (Fig. 1). These results suggest that antimicrobials targeting DNA enhance SCV formation in an SOS dependent manner.

**Fig. 2:**
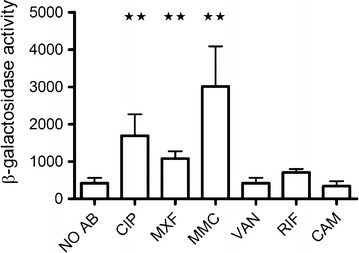
Sub-inhibitory concentrations of DNA-damaging agents activate the SOS-response. Activation of the SOS-response was monitored as β-galactosidase activity from a *recA::lacZ* fusion in strain 8325-4, when grown in absence (NO AB) or in the presence of sub-inhibitory concentrations of CIP (Ciprofloxacin), MXF (Moxifloxacin), MMC (Mitomycin C), VAN (Vancomycin), RIF (Rifampicin) and CAM (Chloramphenicol). The *bars* represent the mean from 3 independent experiments of each condition and error bars represent 95 % confidence intervals

**Fig. 3:**
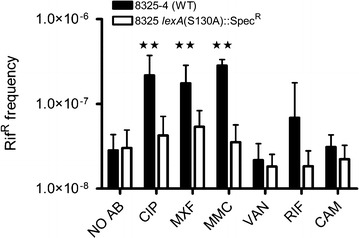
Effect of sub-inhibitory concentrations of antimicrobials on the frequency of rifampicin resistant mutants. The frequency of rifampicin resistant mutants in WT 8325-4 (*black*) population and in the isogenic mutant *lexA(S130A)* (*white*) grown in absence of antibiotics (NO AB) or in the presence of sub-inhibitory concentrations of CIP (Ciprofloxacin), MXF (Moxifloxacin), MMC (Mitomycin C), VAN (Vancomycin), RIF (Rifampicin) and CAM (Chloramphenicol). Concentrations of antimicrobials are 0.5 MIC of the respective strains. The *bars* represent the mean from 8 independent selections of each condition and error bars represent 95 % confidence intervals

**Fig. 4:**
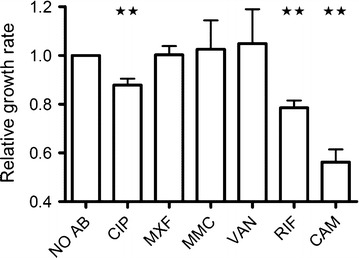
Sub-inhibitory concentrations of antimicrobials on the growth rate of strain 8325-4. The growth rate of strain 8325-4 grown in absence (NO AB) is set to 1 and the relative growth rates are presented of 8325-4 when grown in the presence of sub-inhibitory concentrations of CIP (Ciprofloxacin), MXF (Moxifloxacin), MMC (Mitomycin C), VAN (Vancomycin), RIF (Rifampicin) and CAM (Chloramphenicol). The *bars* represent the mean from 3 independent experiments of each condition and error bars represent 95 % confidence intervals

The increased frequency of SCVs observed in response to compounds causing DNA damage led us to investigate if the SOS regulon was activated. Activation of the SOS regulon was monitored with a *recA::lacZ* fusion in 8325-4. Expression of the *recA* gene was induced when exposed to mitomycin C, ciprofloxacin and moxifloxacin, while none of the remaining three agents increased the expression of *recA* (Fig. 2). The SOS regulon controls the expression of the error-prone polymerase Pol V, which confers increased mutation frequency in *S. aureus* [18]. Reduced replication fidelity could account for the increased number of SCV observed for the agents activating the SOS regulon. Mutation frequency has previously been monitored by rifampicin resistance, which arises by point mutations in *rpoB* [19]. The frequency of rifampicin resistant mutants in un-exposed cultures of the two strains was similar and approximately 3 × 10^−8^. In contrast exposure of 8325-4 to mitomycin C, ciprofloxacin and moxifloxacin increased the frequency of rifampicin resistant mutants 10-, 8- and sixfold respectively, whereas none of the remaining antibiotics for 8325-4 or any of the antibiotics for the *lexA(S130A)* mutant strain affected the frequency of rifampicin resistance (Fig. 3). Several of the antibiotics (ciprofloxacin, chloramphenicol and rifampicin) at the tested concentration also significantly reduced growth rates in exponential phase, yet no correlation between growth rate and increased frequency of SCVs was observed (Fig. 4).

Strains displaying higher basal mutation rates have been detected in several clinical SCV isolates with auxotrophies for hemin [13] and thymidine [20]. Identification of hypermutators among clinical SCV isolates suggests that high mutations rates may contribute to the emergence and establishment of SCV infections [13, 20]. Not only exposure to sub-inhibitory concentrations of antibiotics that trigger activation of the SOS response and a temporary increase in mutation rate may facilitate the emergence of SCVs, but also conditions following contact with the immune system may activate the SOS response [21]. As part of the host defense mechanism against infections, neutrophils generate reactive oxygen species, such as hydrogen peroxide and superoxide [22] that can damage DNA and trigger expression of the SOS regulon [21]. It has recently been shown that exposure to sub-inhibitory concentrations of hydrogen peroxide indeed increases the frequency of gentamicin resistant SCVs via activation of the SOS response [23]. Therefore chemical inhibition that prevents activation of the SOS response could potentially be used as adjuvants to reduce the emergence and establishment of SCVs during infection, a strategy that has also been proposed to decelerate the evolution of antibiotic resistance towards various antimicrobial classes [24, 25].

## Conclusion

In conclusion, our data suggests that environmental stimuli that elevate the mutation rate of *S. aureus*, i.e. through activation of the SOS response, stimulate the emergence of SCVs that are associated with persistent *S. aureus* infections. Caution should be taken when treating *S. aureus* empirically with broad-spectrum antibiotics such as fluoroquinolones when lacking resistance data, since sub-inhibitory exposure could increase the proportion of SCV and thereby limit further treatment options.

## Methods

### Bacterial strains, growth conditions and MIC

The strains used in this study include the *S. aureus* strains 8325-4, 8325 *lexA*(*S130A*)::Spec^R^ [18] and HI2682, a 8325-4 strain containing a *recA::lacZ* fusion [26]. Strains were grown at 37 °C in tryptic soy broth (TSB) or on tryptic soy agar (TSA) plates with or without gentamicin (Sigma), ciprofloxacin (Sigma), moxifloxacin (Sigma), chloramphenicol (Sigma), vancomycin (Sigma), rifampicin (Sigma) and mitomycin C (Sigma). The minimum inhibitory concentrations (MIC) were determined by broth micro-dilution assay according to CLSI guidelines, except that cation-adjusted Mueller–Hinton broth was substituted with TSB.

### Quantification of small colony variants and rifampicin resistant mutants

Over night (ON) cultures of 8325-4 and 8325 *lexA*(*S130A*)::Spec^R^ were diluted 1000-fold in 1 ml TSB in a 10 ml falcon tube, supplemented with or without 0.5 MIC of the antimicrobials assayed. The cultures were grown for 24 h at 37 °C with shaking (200 rpm). Total colony forming units (CFU) were determined on TSA plates, the number of gentamicin resistant small colony variants were estimated on TSA supplemented with gentamicin (4 μg/ml) and rifampicin resistant mutant CFU were determined on TSA supplemented with rifampicin (5 μg/ml). Plates were incubated at 37 °C. Total CFU and rifampicin resistant mutants were counted following 24 h growth and SCVs were counted after 48 h growth. SCVs were defined as colonies resistant to gentamicin (>4 μg/ml) that displayed slow growth and significantly smaller colony morphology (approximately tenfold) compared to WT strain 8325-4. The frequency of SCV was determined as the number of SCV divided by the total CFU count. The same procedure was used in the calculation of rifampicin mutation frequency. The frequencies reported are a mean of 8 independent experiments.

### β-*galactosidase assay with recA::lacZ fusion*

β-galactosidase activity was measured from strain HI2682, a 8325-4 strain with *recA::lacZ* fusion. Cells were grown in 10 ml TSB in 100 ml Erlenmeyer flasks at 37 °C with shaking with and without the presence of sub-inhibitory concentrations (0.5 MIC) of antimicrobials to an OD_600_ of 0.5. The assay was performed as described by Sambrook and Russell [27]. The reported β-galactosidase activities are a mean of three independent experiments.

### Fitness measurement

The fitness of cells exposed to sub-inhibitory concentrations of antimicrobials was estimated by growth rate (µ) comparisons in exponential phase. Growth rates were determined for cells inoculated at 10^6^ cells/ml and grown with or without antibiotics at a concentration of 0.5 MIC at 37 °C in a Bioscreen C (Oy Growth Curves Ab Ltd) with OD_600_ measurements at 5 min intervals. The relative fitness was calculated as: µ(mutant)/µ(WT).

### Statistics

Significant difference was calculated by 1-way ANOVA, using log-transformed datasets for SCV and rifampicin resistance frequency, with a post hoc analysis of Dunnett Multiple Comparison Tests (*p < 0.05, **p < 0.01). Statistics were performed in GraphPad Prism 4 (GraphPad Software, Inc).
